# Disentangling tau: One protein, many therapeutic approaches

**DOI:** 10.1016/j.neurot.2024.e00321

**Published:** 2024-01-25

**Authors:** Courtney Lane-Donovan, Adam L. Boxer

**Affiliations:** Memory and Aging Center, Department of Neurology, University of California, San Francisco, 675 Nelson Rising Lane, Suite 190, San Francisco, CA 94158, USA

**Keywords:** Tau, Neurodegeneration, Alzheimer's disease, Progressive supranuclear palsy, Immunotherapy, Clinical trials

## Abstract

The tauopathies encompass over 20 adult neurodegenerative diseases and are characterized by the dysfunction and accumulation of insoluble tau protein. Among them, Alzheimer's disease, frontotemporal dementia, and progressive supranuclear palsy collectively impact millions of patients and their families worldwide. Despite years of drug development using a variety of mechanisms of action, no therapeutic directed against tau has been approved for clinical use. This raises important questions about our current model of tau pathology and invites thoughtful consideration of our approach to nonclinical models and clinical trial design. In this article, we review what is known about the biology and genetics of tau, placing it in the context of current and failed clinical trials. We highlight potential reasons for the lack of success to date and offer suggestions for new pathways in therapeutic development. Overall, our viewpoint to the future is optimistic for this important group of neurodegenerative diseases.

## Introduction

With the recent FDA approval of the first disease-modifying therapies for Alzheimer's disease, the anti-amyloid immunotherapies lecanemab and aducanumab, we are entering a new era of hope in the treatment of neurodegenerative disease. Though effective in slowing disease or biomarker progression, these treatments still leave much to be desired between their relatively modest clinical effect, risk profile, and limited portion of patients eligible for treatment. Tau protein is implicated in many neurodegenerative diseases, and thus the next phase of promising therapeutics lies in those that target tau protein.

In this review, we introduce the tauopathies and review current knowledge on the complex biology and genetics of tau. We synthesize these concepts with our understanding of tau mechanisms of disease to evaluate the current state of tau therapeutic development. We then discuss why therapeutic successes in nonclinical models have failed to translate to effective treatments and highlight key lessons from the over thirty years of development it took to get to the first FDA-approved amyloid therapy. We finish with a statement on key obstacles in therapeutic development and rollout of tau therapeutics.

## Tauopathies – One Protein, Many Diseases

Most adult neurodegenerative diseases are characterized by the buildup of protein aggregates detectable in the brain at autopsy that are often used for diagnosis. The tauopathies are the subset of neurodegenerative diseases that are associated with the abnormal accumulation of tau protein. Alzheimer's disease (AD) is the most common tauopathy and the most common cause of dementia, affecting over 6 million Americans and many more worldwide [1]. Over twenty tauopathies have been described [2,3]. The tauopathies are categorized by several characteristics, ranging from the tau isoform, aggregate, cell type, and brain region affected. The primary tauopathies are those in which tau pathology is the primary feature and probable driver of disease. Examples include Pick's disease, progressive supranuclear palsy (PSP), corticobasal degeneration (CBD), argyrophilic grain disease (AGD), and globular glial tauopathy (GGT). Secondary tauopathies are those in which tau accumulates in addition to or because of another aggregate or insult. For example, in AD, tau pathology occurs after accumulation of amyloid-beta aggregates. Another example of a secondary tauopathy is chronic traumatic encephalopathy, which occurs after repeated head injury.

Tauopathies are further divided by tau pathology. First, whether they accumulate the shorter 3R isoform of tau, the longer 4R isoform, or a mix of both. Then, which cell type is affected (i.e., neurons or glia), and finally what the appearance of the tau aggregate is to the eye of an experienced neuropathologist (e.g., neurofibrillary tangles, argyophilic grains, etc). Clinically, the symptoms exhibited by a patient are the generally result of the region of the brain impacted by tau, regardless of the underlying pathology. The features of selected tauopathies are summarized in Table 1 and discussed in depth in numerous reviews [2,3].

**Table 1:** Common tauopathies. While over twenty tauopathies have been described, the most common are highlighted above.

Tauopathy	Primary or secondary	Tau isoform	Tau pathology	Clinical Symptoms	Mutations
Pick's disease	Primary	3R	Pick bodies, glial inclusions	Behavioral changes	Sporadic
Corticobasal degeneration (CBD)	Primary	4R	NFTs, coiled bodies, argyophilic threads, astrocytic plaques	Motor, behavioral, visual	Usually sporadic, *MAPT*
Frontotemporal dementia with parkinsonism linked to chromosome 17 (FTDP-7)	Primary	3R, 4R, 3R/4R	Neuronal and glial tau deposits	Behavioral changes, parkinsonism	*MAPT*
Progressive supranuclear palsy (PSP)	Primary	4R	NFTs, tufted astrocytes, coiled bodies	Impaired eye movements, falls, cognitive changes	Usually sporadic, *MAPT*
Primary age-related tauopathy (PART)	Primary	3R/4R	NFTs	Memory impairment	*MAPT H1*
Argyrophilic grain disease (AGD)	Primary	4R	Argyrophilic grains, coiled bodies, pre-NFTs	Cognitive decline, incidental finding	Sporadic
Globular glial tauopathy (GGT)	Primary	4R	Globular astrocytic inclusions, globular oligodendroglial inclusions	Behavioral changes, motor neuron disease	Usually sporadic*, MAPT*
Alzheimer's disease (AD)	Secondary	3R/4R	NFTs	Memory impairment	Usually sporadic, *APP, PS1, PS2, APOE, BIN1,* etc.
Chronic traumatic encephalopathy (CTE)	Secondary	3R/4R	NFTs	Attention and memory impairment, personality changes	*Sporadic*
Down syndrome	Secondary	3R/4R	NFTs	Alzheimer's disease	Trisomy 21 (Triplication of *APP*)
Tuberous-sclerosis complex associated tauopathy	Secondary	4R	NFTs	Behavioral changes	*TSC1, TSC2*

The wide range of clinical presentations, tau isoforms, and mechanisms leading to pathology creates several challenges for effective drug development. How do you identify patients with precision early enough in their disease course? Which part of tau physiology do you target? For diseases without strong genetic links to tau, such as Alzheimer's, is insoluble tau accumulation necessary and sufficient to cause neurodegeneration? To answer these questions, we first must look at tau mechanisms in disease. At the outset of our discussion, it is important to note at this point that it is not definitively clear that tau is causative of disease in all tauopathies, a question that has been amplified by the slow pace of development to date. Thus, the evidence for a causal versus bystander role for tau in tauopathy is addressed throughout the following section.

## Tau in Disease – Same Protein, Multiple Mechanisms

### Tau protein form and function

**Fig. 1:**
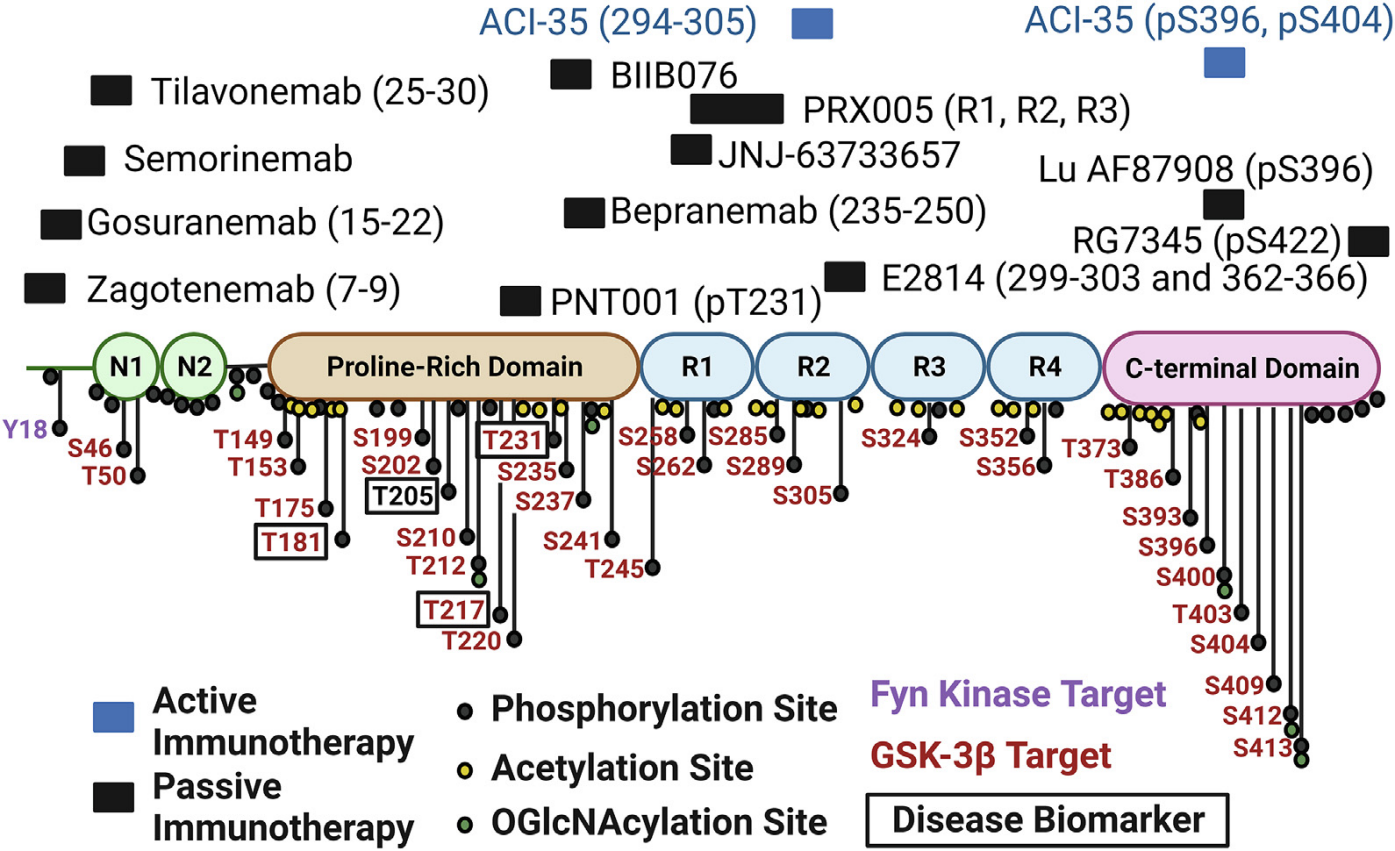
**Structure of tau.** Tau contains two alternatively spliced N-terminal domains, N1 and N2. The proline-rich domain features most clinically developed biomarkers of disease. The microtubule binding region comprises. 4 repeat domains (R1, R2, R3, and R4), of which R2 is excluded in 3R tau. The locations of known or predicted phosphorylation, acetylation, and O-GlcNAcylation sites are shown, with GSK-3β and Fyn kinase targets highlighted. The epitopes, when known, for active and passive immunotherapies against tau are shown. Created with BioRender.com.

Tau has multiple roles in brain function, of which the most prominently described is microtubule binding, which stabilizes microtubule structures and promotes a variety of cellular functions, including neurite outgrowth [4,5]. Tau contains specialized domains that each have unique roles and binding properties: the N-terminal projection domain, the proline-rich region, the microtubule binding repeat (MTBR) domain, and the C-terminus. Fig. 1. The human tau protein primarily exists in 6 different isoforms, depending on the number of repeats in the N terminal domains (0 ​N, 1 ​N, or 2 ​N) and the number of MTBR domains (3R or 4R) [6]. This difference derives from the inclusion or exclusion of exon 2 or 3 (0, 1, or 2 ​N) or exon 10 (3R or 4R) [7]. Fetal tau is predominantly 3R tau, while in adulthood, expression is closer to 1:1 between 3R and 4R [8]. Tauopathies can then be subdivided by the tau isoform that accumulates: 3R (e.g., Pick's), 4R (e.g., PSP), and mixed 3R/4R (most secondary tauopathies).

There are two other tau isoforms that are relatively understudied. “Big tau”, encodes exon 4a, resulting in an additional ∼250 amino acid sequence shortly after the N terminal domain [9,10]. Big tau is predominantly expressed in the adult peripheral nervous system and certain neurons of the cerebellum [11]. Another isoform encodes exon 6 and is found in spinal cord and skeletal muscle [12]. Very little is known about these isoforms, though they may impact microtubule binding and neurite outgrowth [13,14]. Typically, residues in tau are identified according to the 441 amino acid sequence of 2N4R tau, not the 733 amino sequence of big tau, and we follow this standard nomenclature in this article.

### Tau genetics

Tau protein is encoded by the *MAPT* gene at chromosome 17q21-22. A variety of mutations within both the *MAPT* coding and noncoding regions affect tau function and disease risk. Over 60 mutations in *MAPT* have been definitively linked to disease, with many more variants of unclear pathogenicity identified [15]. For an up-to-date list of all identified tau mutations, please see (https://www.alzforum.org/mutations/mapt). All are believed to be gain of function, with no loss of function mutations identified to date. Most mutations in tau are linked to FTLD-tau with bvFTD clinical phenotypes [16]. Mutations in the coding region of tau have been shown to have a variety of effects and can modulate tau aggregation, cleavage by lysosomal proteases, microtubule binding and assembly, and the ability of tau to be phosphorylated through the introduction or removal of phosphorylation sites [[17], [18], [19], [20], [21], [22], [23]]. A subset of *MAPT* mutations have been identified, such as IVS10+16, that result in phenotypes that are more clinically similar to PSP or CBD, which are generally considered sporadic diseases [24]. The PSP *MAPT* mutations generally promote inclusion of exon 10, thus shifting tau production toward the 4R isoform and generating a 4R tauopathy [25].

SNP analysis of tau revealed two distinct extended haplotypes of the *MAPT* locus, H1 and H2. These haplotypes result from an ancestral inversion of 900 ​kb at 17q21 that covers a region of the chromosome that contains not just *MAPT*, but at least 5 other gene loci. The H1 haplotype is significantly overrepresented in patients with PSP, CBD, FTD, and Parkinson's disease relative to the less common H2 haplotype, which may be protective against disease [[26], [27], [28]]. A role for tau haplotypes in AD is less well defined, and H1 appears to have an effect on disease risk only in individuals who lack a copy of the ApoE4 allele, the most common genetic risk factors for AD [29]. The description of this haplotype has been expanded over the years to include many sub-haplotypes, prompting investigation into whether the other genes encoded in the haplotype may be relevant to disease [30]. For example, PD risk is increased in an H1 sub-haplotype that has altered copy numbers of *LRRC37A*, another gene within the locus, and thus may be completely independent of *MAPT* [31].

Overall, the existence of numerous *MAPT* mutations is considered the strongest evidence for a causal role of tau in neurodegeneration. Thus, it is important to note that a tau mutation has never been identified to cause AD, while many mutations in the amyloid precursor protein *(APP)* and presenilin genes (*PSEN1&2*) have been found for autosomal dominant familial AD [32]. This has led to open debate on whether tau is causal in AD or simply a byproduct of the underlying disease process. The strongest argument for tau as causal in AD is the finding that the areas of tau deposition on PET and postmortem pathology correlate more strongly to symptom onset than amyloid [33]. Also, two tau mutations, R406W and V337 ​M, that produce 3R:4R tau similar to AD sometimes produce a clinical amnestic syndrome resembling AD, but without beta amyloid deposition [6,7]. Finally, genetic deletion of tau in mouse models of AD abrogates clinical phenotypes. Thus, the question may not be if tau is involved in AD, but rather when in disease course a therapeutic would be effective.

### Tau post-translational modifications

After gene expression and translation, proteins can be altered by numerous mechanisms that affect their activity, localization, and processing in the cell, and tau protein is no exception. These post-translational modifications (PTMs) range from phosphorylation (of which tau has over 60 potential sites), acetylation, glycosylation, O-GlcNACylation, and cleavage, among others. Tau is modified by all these, reviewed in depth in Ref. [34], and briefly summarized here.

The accumulation of hyperphosphorylated tau is a key pathological finding in most tauopathies, thus it is the best studied of the PTMs [35]. Phosphorylation can occur on threonine, serine, and tyrosine residues and effectively adds a negative charge to a protein, which can affect both structure and function. Phosphorylation of tau on the MTBR modulates binding of tau with the microtubule, while phosphorylation of some sites promotes tau aggregation [36]. Most of the identified tau clinical biomarkers feature phosphorylation sites in the proline-rich domain, and the mechanistic effect of this phosphorylation is unclear [37]. Not every residue is deleterious if phosphorylated. For example, phosphorylation at S305 prevents tau aggregation, an effect that is lost in the S305N *MAPT* mutant [38]. Some drugs in early clinical development for tau target its hyperphosphorylation. These include GSK3β inhibitors and Fyn kinase inhibitors, described in detail in a later section.

Additional therapeutics in development are directed against acetylation and O-GlcNACylation. Tau is not only hyperphosphorylated in disease, but also hyperacetylated. Acetylation of tau stabilizes the formation of tau fibrils and promotes aggregation of 3R tau [39,40]. Moreover, tau acetylation blocks its degradation through chaperone-mediated autophagy and routes it to extracellular release [41]. O-GlcNAcylation is the addition of N-acetylglucosamine to serine and threonine residues, which physically blocks the ability of the residue to be phosphorylated. O-GlcNAcylation of tau is decreased in AD brain extracts [42].

Some post-translational modifications do not currently have therapeutics in development but are worth touching on here. Glycosylation is the addition of a carbohydrate to a protein and the most common post-translation modification. In AD brain, tau is abnormally overglycosylated, which may promote phosphorylation [43]. Tau cleavage is another major PTM of tau. In disease, tau can be cleaved at numerous sites resulting in the production of a variety of fragments, and both N- and C-terminal fragments have been found to be pathologic [44]. The region that forms disease-associated insoluble aggregates is close to the C-terminus, and thus C-terminal fragments may be more prone to aggregation [45]. On the other hand, N-terminal fragments are secreted into the CSF where they are synaptotoxic, and phosphorylation on these N-terminal fragments forms the basis of most biomarker development [[46], [47], [48]].

An important finding is that PTMs can vary depending on the tauopathy [49]. This provides both an opportunity and a challenge for biomarker and therapeutic development. While divergent PTMs may allow the precise development of a disease-specific biomarker or therapeutic, it could limit the ability to translate some therapeutics between tauopathies.

### Tau aggregation

The accumulation of tau aggregates within the cell is a key feature of disease pathology. Short amyloid-forming motifs at the beginning of each of the 4 repeats (i.e., R1, R2, R3, and R4) control tau fibril assembly by interacting with other parts of the protein [50]. Disease-associated mutations in tau are often located within these domains and promote fibril aggregation. Tau fibrils exhibit a prion-like capacity to seed and induce fibril formation in normal tau, thus promoting transsynaptic spread between neurons [51]. Fascinatingly, the fibrils derived from the brains of different tauopathies have distinct structures, and these unique structures are conserved after seeding [52]. Thus, a therapeutic that impacts formation or spread of one species of tau fibril, but not another, may only be effective in that disease [53]. It is unclear if it is a toxic gain of function of aggregated tau or a loss of normal tau function that drives disease.

### Tau degradation

Protein homeostasis, or proteostasis, is carefully maintained by the cell, and breakdown of proteostasis leads to the accumulation of misfolded and aggregated proteins such as tau. Misfolded or aggregrated tau can be cleared from the cell by three mechanisms: ubiquitination and degradation via the proteosome, autophagy, or release by exosomes [41,44,54]. The latter mechanism is hypothesized to contribute to the transsynaptic spread of tau between cells. Promoting degradation of tau via ubiquitination or autophagy is relatively underexplored avenue for therapeutic development; however, preclinical development of PROTACs targeting tau to the proteosome is underway [55]. Genetic evidence in both primary and secondary tauopathies suggests that ubiquitin-mediated proteosomal clearance of tau may be an important factor in mitigating the effects of tau pathology [56,57].

## Human Biomarkers of Tauopathy

Clinical biomarkers to detect tau pathology have been reviewed in depth elsewhere, thus we only briefly touch on them here [58]. Broadly, tau-PET tracers reflect tau deposition in AD brain; however, the tracers typically become positive later in disease stage. Additionally, in other tauopathies, tau-PET is not accurate in diagnosing disease, though work is ongoing to identify tau tracers for these diseases [59]. Plasma and CSF biomarkers tend to be positive much earlier in disease. For AD, ptau181, ptau217, and ptau231 are under active development and more closely mirror the arrival of amyloid pathology, an earlier timepoint in disease. Clinical testing for ptau181 and ptau217 is beginning to be used diagnostically for AD. Interestingly, for PSP, it may not be tau but rather neurofilament light chain (Nfl), a general marker of neurodegeneration, is more useful as a diagnostic biomarker [60].

## Tau-Directed Therapeutics: Lack of Success to Date, but Reasons for Optimism

To date, no tau-directed therapeutic has gained FDA approval for the treatment of any neurodegenerative disease or has been shown to have an impact on clinical measures of disease progression. Broadly, most current therapeutic strategies fall into three mechanistic categories: those directed at post-translational modifications, those directed at impacting tau function or aggregation and those that aim to reduce tau levels overall. Most tau therapeutics have been tested for the treatment of AD or PSP. The reasons for the selection of these diseases are multifold. As the most common dementia, there are many AD patients who are interested in participating in trials, thus making it easier to achieve enrollment goals. Biomarkers are also the most developed for AD, thus facilitating monitoring of biologic target engagement and therapeutic response in lieu of classical clinical outcome measures, which can help refine and speed up trials. These large patient numbers and precise biomarkers make it possible to overcome the variability of clinical phenotype and underlying pathology seen in the MCI and early AD patients that are usually recruited to these trials. Conversely, though PSP has far fewer patients to enroll in trials and fewer diagnostic and response biomarkers, they are clinically distinct from other neurodegenerative diseases, follow an expected disease course, and almost always have 4R tau pathology on autopsy. This is in stark contrast to CBS, bvFTD, and PPA, which can have a wide variation in both clinical phenotype and underlying pathology [61]. With ongoing biomarker development to differentiate between pathology in these diseases, more clinical trials will engage those patient populations.

**Fig. 2:**
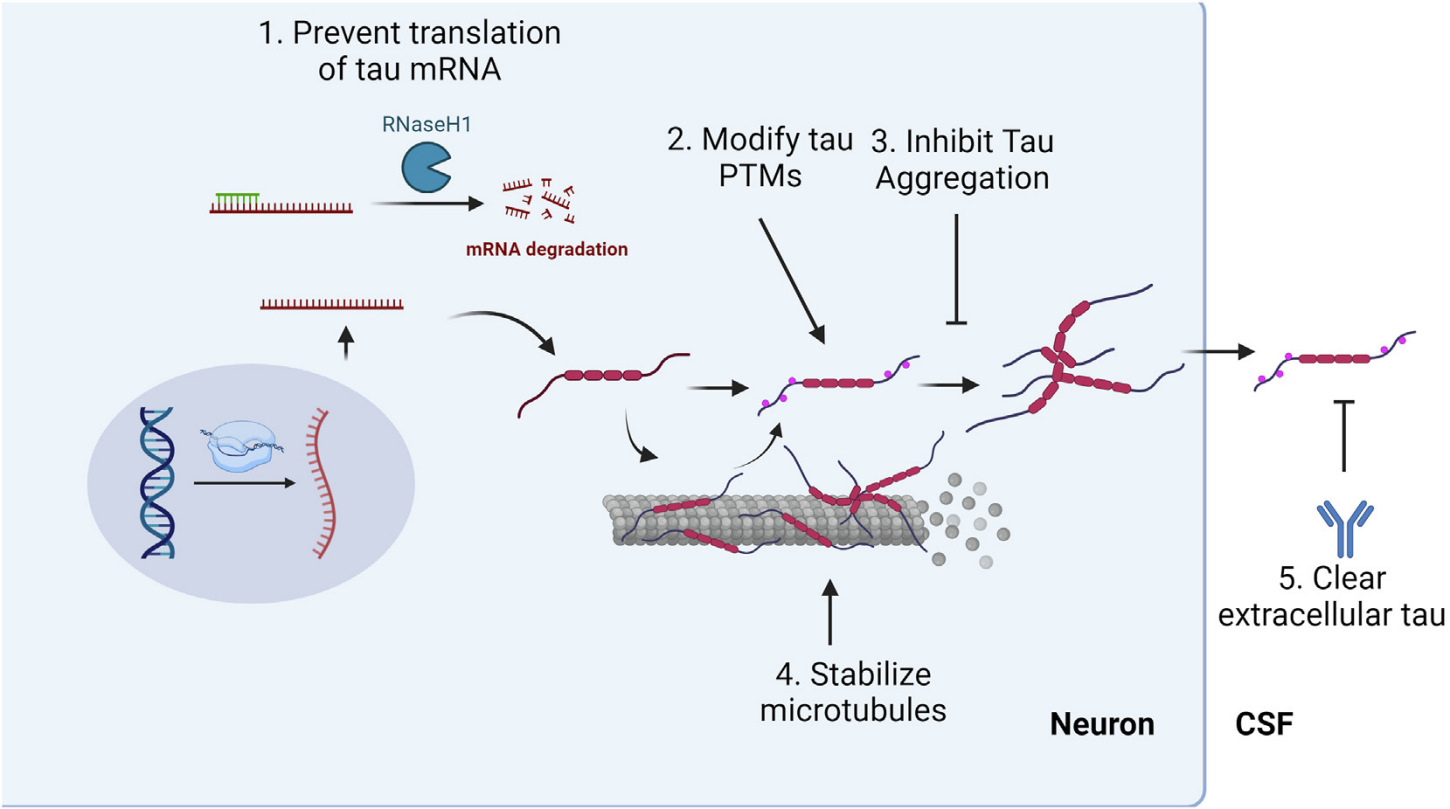
**Mechanism of therapeutic action against tau.** Tau levels can be reduced 1) at a translational level by ASO or 5) cleared extracellularly via active or passive immunotherapy. 2) Tau PTMs, such as phosphorylation, acetylation, or O-GlcNAcylation, can be modulated via small molecules. 3) Tau aggregation can be inhibited. 4) A lost tau function can be restored with microtubule stabilizers. Created with Biorender.com.

In this section, we focus on the main ongoing and discarded therapeutic strategies to alter tau in disease. Fig. 2. For each therapeutic class, we provide the targeted disease mechanism, selected nonclinical data to support intervention, and prior and ongoing therapeutic trials. Table 2. Where appropriate, we highlight potential causes of failure.

**Table 2:** Ongoing and completed clinical trials in tau-directed therapeutics.

Agent	Mechanism	Disease target	Phase	Trial identifier	Status
Lithium	Anti-GSK-3ß	PSP, CBS, MCI	I/II	NCT00703677, NCT03185208	Negative, ongoing
Valproate	Anti-GSK-3ß	PSP	II	NCT00385710	Negative
Tideglusib	Anti-GSK-3ß	Mild-moderate AD, PSP	II	NCT01350362, NCT01049399	Negative
Saracatinib	Fyn kinase inhibitor	Mild AD	II	NCT02167256	Negative
LY3372689	OGA inhibitor	AD	II	NCT05063539	Ongoing
BIIB113	OGA inhibitor	Healthy volunteers	I	NCT05195008	Ongoing
ASN51	OGA inhibitor	Healthy volunteers, AD	I	NCT05725005, NCT04759365	Ongoing
ASN90	OGA inhibitor	PSP	I	Unlisted	Ongoing
Salsalate	Acetylation inhibitor	PSP, mild-moderate AD	I/Ib	NCT02422485, NCT03277573	Negative
TRx0237	Tau aggregation inhibitor	bvFTD, AD	III	NCT01626378, NCT01689246	Negative
OLX-07010	Tau aggregation inhibitor	Healthy volunteers	I	NCT05696483	Ongoing
Davunetide	Microtubule stabilization	PSP, MCI	II/III	NCT01110720, NCT00422981	Negative
Abeotaxane	Microtubule stabilization	AD, 4R tauopathies	I	NCT02133846, NCT019666666	Worse outcomes
Epothilone D	Microtubule stabilization	Mild AD	I	NCT01492374	Discontinued
BIIB080	Tau ASO	Mild AD	II	NCT05399888	Ongoing
NI0752	Tau ASO	PSP, mild AD	I/Ib	NCT04539041, NCT05469360	Ongoing
AADvac1	Tau-directed vaccine	AD, nfPPA	I/II	NCT02579252, NCT03174886	Negative, ongoing
ACI-35	Liposomal vaccine	Mild AD	I/II	NCT04445831	Ongoing
Gosuranemab	Tau N-terminal antibody	PSP, mild AD, CBS	II	NCT03068468, NCT03658135, NCT03352557	Negative, worse
Tilavonemab	Tau N-terminal antibody	PSP, AD	II	NCT03712787, NCT03413319, NCT02985879	Negative
Zaqotenemab	Tau N-terminal antibody	AD	II	NCT03518073	Negative
Semorinemab	Tau N-terminal antibody	AD	I/II	NCT03828747	Negative
BIIB076	Mid-domain tau antibody	Mild AD	II	NCT03056729	Discontinued
E2814	Mid-domain tau antibody	Familial AD	II	NCT04971733	Ongoing
JNJ-63733657	Mid-domain tau antibody	Mild AD	II	NCT04619420	Ongoing
Bepranemab	Mid-domain tau antibody	AD, PSP	II	NCT04867616, NCT04658199	Ongoing
PRX005	Mid-domain tau antibody	Healthy participants	I	Unlisted	Ongoing
Lu AF87908	C-terminal ptau antibody	AD	I	NCT04149860	Completed
PNT00I	C-terminal ptau antibody	AD, TBI	I	NCT04096287, NCT04677829	Ongoing
RG7345	C-terminal ptau antibody	Healthy participants	I	NCT02281786	Discontinued
APNmAb005	Tau oligomer antibody	Healthy participants	I	NCT05344989	Ongoing

### Tau PTM-directed therapeutics

#### Inhibitors of tau phosphorylation

GSK-3β phosphorylates numerous residues on tau, including several that have been identified as biomarkers of disease. GSK-3β activity is increased in Alzheimer's disease brain, and blocking its activity has had ameliorating effects in AD mouse models [62]. The multitudinous roles of GSK-3β in the CNS were recently reviewed in Ref. [63] As a result of these findings, several GSK-3β inhibitors have been repurposed or developed for early to mild AD.

Lithium has several targets, including inhibition of GSK-3β. One clinical trial showed lithium slowed progression in patients with mild cognitive impairment [64]. A trial of lithium in PSP and CBS was stopped early due to falls, a known side effect of lithium that may limit its clinical utility. Valproate, an anti-seizure medication, was also assessed in PSP because of anti–GSK-3β activity, but it did not slow decline in PSP Rating Scale (PSPRS) scores [65].

Since lithium and valproate both affect targets other than GSK-3β, a GSK-3β specific inhibitor, tideglusib, was developed. Tideglusib did not slow progression in either mild to moderate AD or PSP [66,67]. However, the tideglusib AD trial did potentially identify a subgroup with mild AD that received a lower drug dose might have had slowing of disease. Though further trials in AD are not currently being pursued, tideglusib is currently being studied in ALS and myotonic dystrophy (ClinicalTrials.gov NCT05105958, NCT05004129). Other inhibitors of GSK-3β with different structural mechanisms of inhibition are in preclinical development [68].

Another tau kinase is Fyn kinase, from the src family tyrosine kinase (SFK) family. Unlike GSK-3beta, which phosphorylates numerous sites in tau protein, Fyn kinase phosphorylates only one tau residue, tyrosine 18 (Y18) [69]. The primary role of Fyn *in vivo* is regulation of signal transduction at the synapse [70,71]. Fyn has tau-dependent and tau-independent roles in neurodegenerative disease that make it an appealing therapeutic target [72]. Independent of tau, amyloid-β oligomers interact with cellular prion protein (PrP^c^) at the synapse to activate Fyn signaling, which results in downstream changes in NMDA receptor signaling, dendritic spine structure, and tau phosphorylation [73]. Tau modulates Fyn kinase activity by trafficking Fyn to the synapse, and blocking this activity of tau ameliorates amyloid-beta derived synaptic toxicity [74]. Inhibition of Fyn in the P301S tauopathy mouse model rescued memory deficits and tau accumulation [72]. Thus, inhibition of Fyn could ameliorate 1) deleterious effects of amyloid on synaptic function, 2) amyloid-independent synaptic toxicity, and 3) downstream tau phosphorylation. Only one Fyn kinase inhibitor has gone to clinical trial, AZD0530, a repurposed cancer drug. AZD0530 was ineffective in a phase IIa trial for mild Alzheimer's disease and was also noted to have significant GI side effects [75]. An alternative approach currently in preclinical development is to inhibit the interaction between Tau and the SH3 domain of Fyn [76].

#### Other PTM modifiers

OGA is the enzyme that removes O-GlcNAc from proteins, thus inhibitors of OGA are in development to promote tau O-GlcNAcylation. OGA inhibitor treatment in P301L models of tauopathy reduced tau phosphorylation, aggregation, and neuron loss [77]. A phase II trial with the OGA inhibitor LY3372689 in early AD is currently underway. The development of this drug has been supplemented with the co-development of a PET ligand, LSN3291920, that has enabled the assessment of OGA enzyme occupancy of a ligand [78]. Using this tracer, the company has demonstrated up to 90 ​% of occupancy of brain OGA by the drug in humans subjects [79]. Inhibition of OGA would be expected to impact many proteins that are O-GlcNAcylated, not just tau, and the results of that trial should be interpreted with this in mind when they become available. As an example, an OGA inhibitor blocked aggregation of an unrelated protein, TAK-1 binding protein, and O-GlcNAcylation on synuclein aggregation is actively being explored for Parkinson's disease [77,80]. Other OGA inhibitors in phase I include BIIB113, ASN51, and ASN90.

In addition to hyperphosphorylation, tau becomes hyperacetylated at numerous residues in disease. Salsalate is an NSAID that also inhibits acetylation, but it was unfortunately not well tolerated in a phase I trial in PSP and AD. A more directed inhibitor of tau acetylation might be promising, given the effect of acetylation on tau aggregation and degradation; however, none are currently in clinical development. Overall, a limitation on the drugs that address tau PTMs is that they would be expected to broadly affect the PTMs of many proteins, which may cause substantial off-target effects.

### Tau function and dysfunction-directed therapeutics

#### Microtubule stabilization

Hyperphosphorylation of tau limits its ability to bind to microtubules and promote their stability. Several microtubule stabilizers that were initially developed for chemotherapy for cancer have been repurposed to compensate for this lost function of tau in the CNS.

Davunetide is a peptide salt based on activity-dependent neuroprotective protein (ADNP) that is given intranasally. In nonclinical models, davunetide promoted microtubule stability and reduced tau phosphorylation [81]. A phase II/III trial in PSP patients did not show an effect on decline in PSPRS [82]. A phase II trial in patients with MCI showed that it was well tolerated with a potential signal on efficacy; however, the compound was not pursued further [83]. Another microtubular stabilizer, epothilone D, improved cognitive performance in the PS19 tauopathy mouse model, even when the compound was given after the development of tau tangles and cognitive deficits had already occurred [84]. This compound did not progress past phase I. The final compound in this class, abeotaxane, was tested in a basket trial design in patients with AD and 4R tauopathy (CBS and PSP). Three patients in the AD group had severe anaphylactoid reactions, and more falls were noted in the AD treatment group compared to placebo, an interesting disease-specific response to the treatment [85]. Despite promising results in nonclinical trials, the microtubule stabilizers have largely been dropped due to lack of clinical effect.

#### Tau aggregation

Methylene blue is an old malaria drug that was found to block tau aggregation and thus was developed into TRx0237 for the treatment of tauopathy [86]. Several phase III trials in the drug were negative [87]. However, these studies used a low dose of the drug, which stains urine blue, as placebo, and both placebo and treatment groups progressed more slowly than expected [87]. Thus, redesigned trials using the low dose and a different placebo have been proposed. OLX-07010 is a small molecule that was identified in high-throughput screen for compounds that block the self-association of full-length, non-mutated tau. In nonclinical models, OLX-07010 reduced tau hyperphosphorylation in htau mice [84]. The compound will start phase I trials in healthy volunteers this fall.

### Therapeutics that reduce tau

#### Biologics

The largest class of tau-directed therapeutics in development are the passive immunotherapies. These are biologic, humanized antibodies raised against specific epitopes of the tau protein, with the goal of lowering tau levels. The first subclass to reach clinical development were passive immunotherapies directed against the N-terminal domain of tau: gosuranemab, tilavonemab, zagotenemab, and semorinemab. Phase II trials have been largely without clinical benefit despite evidence of reduction of CSF N-terminal tau. In one trial of mild-to-moderate AD, a slightly more severe subgroup than the other trials, semorinemab led to a 43.6 ​% slowing of decline on the Alzheimer's Disease Assessment Scale-Cognitive Subsale (ADAS-Cog), without benefit on other outcomes [88].

There are a few hypotheses for why passive immunotherapy against the N-terminal is ineffective. N-terminal tau accumulates in the CSF, but may represent the end stage of a clearance pathway into the CSF. In a PSP phase II trial, gosuranemab was effective in reducing CSF N-terminal CSF; however, 3 patients who went to autopsy showed no change in intracellular tau pathology, although there was evidence of tau accumulation in the lysosomes of perivascular astrocytes [89,90]. Moreover, the amyloid forming region of tau is in the C-terminal MTBR, thus persistence of that fragment may still cause disease pathogenesis. Failures in N-terminal directed tau antibodies has led to the development of antibodies against epitopes in the proline-rich domain (BIIB076, bepranemab), the MTBR (E2814, JNJ-63733657, PRX005), the phosphorylated C-terminal domain (Lu AF87908, RG7345), and oligomeric tau (APNmAb005). One unique antibody is PNT001, developed against the cis phosphorylated T231 residue, which acutely rises post brain injury and is resistant to dephosphorylation and degradation [91,92]. This antibody was planned to be tested in both acute traumatic brain injury (TBI) patients and AD; however, it was terminated (NCT04677829). Most of the trials in tau passive immunotherapies are ongoing. The epitopes of the antibodies are depicted in Fig. 1 [93]. An overall concern with this class is that depleting extracellular tau may not significantly affect intracellular tau or be sufficient or early enough in disease to prevent transsynaptic spread.

#### Vaccines

With the identification of amyloid protein in Alzheimer's disease, initial clinical development efforts focused on a vaccine directed against the amyloid-β peptide. Unfortunately, the first anti-amyloid vaccine to be developed caused fatal encephalitis in 6 out of 300 immunized patients in the early 2000s [94]. Subsequently, enthusiasm for active immunotherapy for dementia temporarily waned; however, advances in our understanding of the autoimmune reaction that led to that poor outcome has guided the development of further vaccines. There are now two vaccines against tau in clinical development for neurodegenerative diseases. The first, AADvac1, is derived from a small peptide within the MTBR domain of tau coupled to keyhole limpet hemocyanin and uses an aluminum hydroxide adjuvant. In nonclinical rat models, the vaccine reduced hyperphosphorylated tau, tau oligomers, and improved memory [95]. In a phase II trial in mild AD patients, AADvac1 slowed the increase of plasma Nfl, but it did not impact cognitive outcomes overall [96]. A post-hoc sub-study suggested that a certain subset of younger patients may benefit, and the company plans on future stratified trials to better evaluate clinical efficacy. A phase I trial of AADvac1 in nfPPA is ongoing. ACI-35 is a liposome-based vaccine that contains synthetic tau fragments phosphorylated at the residues S396 and S404. In nonclinical wildtype and P301L mutant mice, treatment with the vaccine starting shortly after birth raised antibodies against phosphorylated tau only and rescued some behavioral phenotypes [97]. After some redesign of the vaccine in phase I to increase immune response, the company plans to move into phase II.

#### ASOs and gene therapies

The first antisense oligonucleotide developed for a CNS indication was nusinersen for spinal muscular atrophy, a rare genetic disorder that causes progressive motor weakness and ultimately death. Nusinersen was a striking clinical success, starting a new genetic revolution in our approach to the treatment of rare diseases [98]. The ASO tofersen was recently approved by the FDA for the treatment of ALS due to *SOD1* mutations, despite only showing improvement in biomarkers and not clinical outcome, highlighting the confidence of the FDA and drug developers in biomarkers for neurodegenerative disease [99]. Briefly, an ASO is a single-stranded deoxyribonucleotide that is complementary to the mRNA target. Most ASOs induce RNase H endonuclease cleavage of the RNA-DNA heteroduplex, reducing the translation of the target gene product [100]. In nonclinical mouse and nonhuman primate (NHP) models, tau ASOs reduced preexisting phosphorylated tau, neurodegeneration, and behavioral defects [101]. Two ASOs directed against tau have reached the clinical stage of development. In a phase I trial for BIIB080, tau ASO successfully reduced CSF pTau181 by up to 50 ​%. Excitingly, this ASO was also reported to partially reduce insoluble tau measured by MK6240 PET in a small number of patients in the Phase 1 study [102]. Moreover, the company recently presented results showing a potential clinical benefit after 100 weeks of treatment [103]. The most common side effects (headache, nausea) were related to the lumbar puncture required to deliver the ASO [104]. A phase II trial is currently enrolling. NIO752 is another tau ASO that is being pursued for PSP and mild AD in phase I trials.

## From Mouse to Man: Limitations in Translation

Why haven't there been better outcomes from tau therapeutic trials to date? The answer may lie in several sources: limitations in nonclinical models, incorrect identification of target, and inability to engage target.

First, though there are many ways to investigate tau biology in a laboratory setting, each has its flaws. Common *in vitro* tissue culture models range from non-CNS derived cells (e.g, HEK293 line, derived from embryonic kidney) to neuron-like (e.g., SH-SY5Y, a neuroblastoma line) to iNeurons derived from induced pluripotent stem cells and acutely prepared mouse embryonic neurons. Each cell line has its own set of advantages and disadvantages, and all have transcriptional signatures of a fetal cell, thus limiting the ability to study the effect that aging, or even more than a few weeks in culture, has on disease pathology. Newer methods attempt to circumvent limitations of *in vitro* culture. Direct transdifferentiation of skin fibroblasts to neurons maintains the aging transcriptional signature, while 3D organoids and co-cultures with glia attempt to recapitulate some of the cell-cell signaling that impacts disease [105,106].

In contrast to tissue culture, animal models such as mice offer a way to study neurodegeneration in the context of both aging and the cellular milieu of the CNS. Unfortunately, there are some substantial differences in the signatures of aging between mice and humans, and even differences between mouse tau and human tau [107]. As an example, mouse models that overexpress human *APP* harboring familial AD mutations generally do not develop tau pathology unless human *MAPT* FTLD mutations are also present [108]. Thus, the tau studied in those models represents an FTLD tau, not an AD tau. Similarly, there are numerous models in which mice are “seeded” with pre-formed fibrils that induce aggregation of native tau. As described above, tau fibrils vary by disease type, which means that many of the nonclinical models tested for therapeutics may not be appropriate for the intended disease.

Many nonclinical studies initiate treatment well before symptom onset in mice, which is not clinically feasible for patients with the currently available biomarkers. When treatment starts after symptom onset, what is monitored? Lithium treatment in mouse models did not reverse pre-established NFTs despite lowering hyperphosphorylated tau [109]. This raises concern for our clinical trials – if a reduction plasma and CSF tau biomarkers do not reflect intracellular change in pathology, it limits our ability to interpret trial failures. A new CSF MTBR biomarker seems to be more sensitive to the accumulation of insoluble tau in AD and may be more useful than first generation tau fluid biomarkers [46].

### Lessons from amyloid

An FDA-approved anti-amyloid therapy for AD took over 20 years of repeated disappointments in clinical development. Several obstacles had to be overcome. First, the correct population to treat had to be determined. Early studies in moderate AD were negative. This led to the theory of the “ca-tau-strophe”, a point at which amyloid pathology is superseded by tau as the primary driver of disease and amyloid therapies are no longer effective [110]. Subsequently, recruitment focused on patients earlier in clinical disease course. Second, sensitive and specific biomarkers had to be developed (CSF amyloid and tau, amyloid PET tracers) to determine which patients with MCI or clinical AD had true AD pathology and to evaluate whether anti-amyloid treatments effectively cleared amyloid from the brain. These two challenges in drug development took decades of constant refinement.

Drug developers in the CNS space have quickly learned from the amyloid therapies. Effectively all trials now incorporate CSF and/or PET biomarkers in addition to clinical scales to accelerate drugs through phase II quickly without requiring a clear change on clinical measures. Moreover, companies are discovering and designing novel biomarkers, like the OGA PET ligand described above, to incorporate into their trials to confirm target engagement. Many of these emerging biomarkers will ultimately be utilized in clinical practice to determine appropriate patient selection and to follow treatment effect.

## Vision of the Future: Current Questions and Moving Forward

The FDA approval of disease-modifying therapies for two neurodegenerative diseases has re-invigorated excitement for the future and highlighted the need for faster, more precise trials in neurodegeneration. Optimizing each step, from initial preclinical discovery to phase III clinical trial, is necessary to bring more interventions effectively and quickly to patients.

At the beginning, before drugs are brought to humans, thoughtful design and interpretation of the nonclinical studies is required. Is the targeted mechanism causal, or is it simply a byproduct of disease? Is the right tau strain being studied? Are tau strains a cause or byproduct of human disease? Is the intervention given at a timepoint that is clinically relevant? If the answer to any of these is no, caution may be warranted in decisions to advance the therapy to the clinic. Ultimately, none of the animal or cell culture models of AD or tau pathology, except for the *APP* and other similar beta amyloid overexpressing transgenic mice that were used to develop the first anti amyloid vaccine (AN1792), have been predictive of treatment effects in humans. Therefore, once a convincing biochemical efficacy signal is observed in a relevant mouse model, it may be best to move the drug to the clinic for further testing if it has a favorable safety profile and promising mechanism of action.

**Fig. 3:**
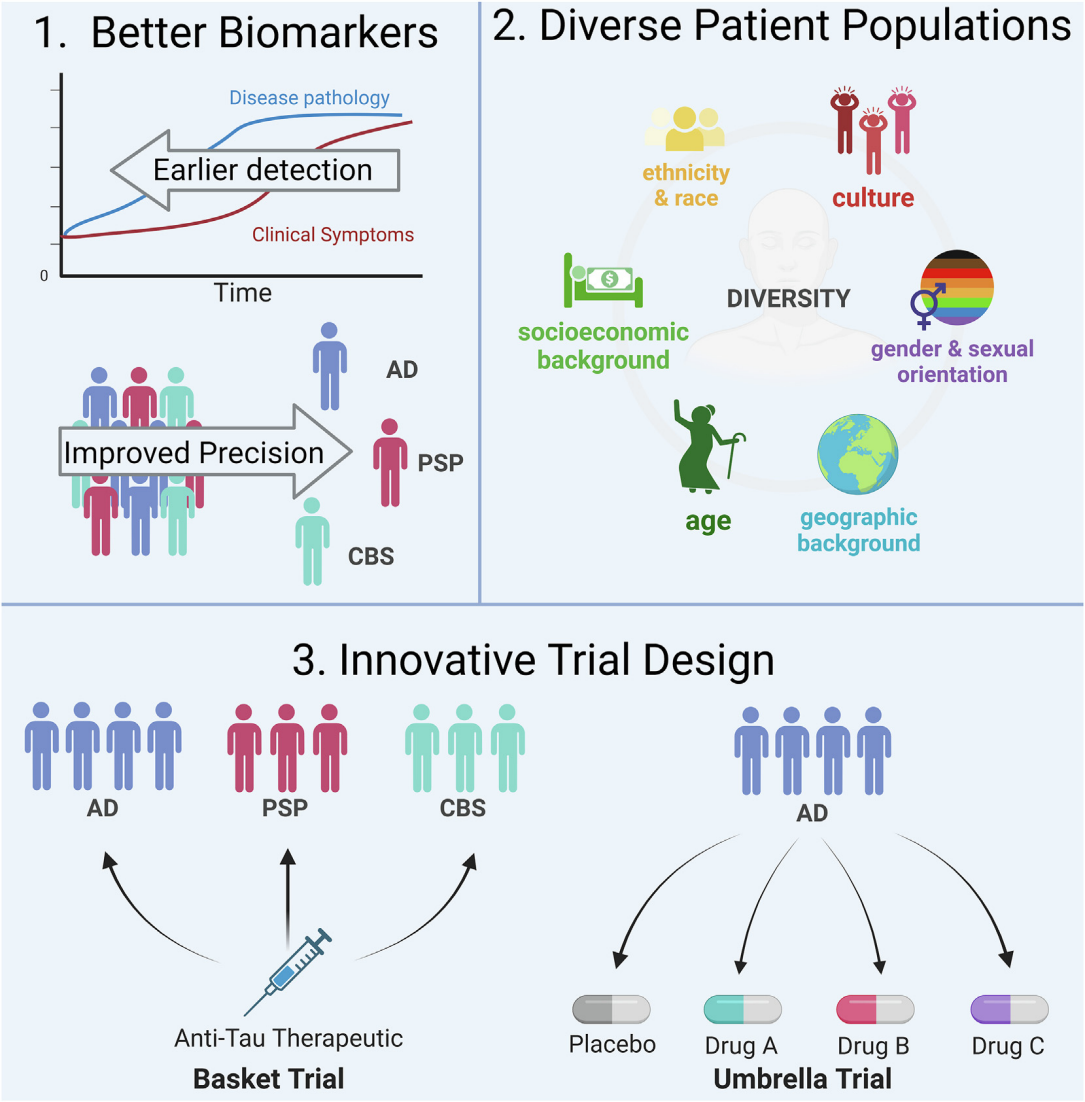
**Methods to improve outcomes in clinical trials.** 1. Better biomarkers that can accurately detect specific diseases early in disease course. 2. Increased representation of groups that have been historically underrepresented in clinical trials. 3. Use of innovative and efficient trial design to improve power. Created with BioRender.com. Section two adapted from “Why We Need to Increase Diversity in the Immunology Research Community” from BioRender.com.

Next, to effectively run clinical trials for neurodegeneration, there are several important obstacles to overcome. First, we must continue to develop biomarkers that are accurate, allow early diagnosis, and discriminate between pathologies. Second, it is well established that clinical trials disproportionately enroll white, affluent participants. If the goal of drug development is to treat all affected patients, then trial design and recruitment must focus on enrolling patients that accurately reflect the intended patient population. Finally, with only a limited number of patients to enroll and many therapeutics to test, particularly for rare diseases like PSP, innovative and efficient trial designs are necessary. For example, an umbrella trial design allows the use of one placebo group against several intervention groups, thus increasing the number of patients who can receive a trial drug relative to placebo and reducing the cost and time necessary to test each individual treatment. Basket trials, taken from cancer research, allow the use of a single therapeutic in different diseases that share a pathology (i.e., tau) [111,112] (Fig. 3).

Finally, a looming question must be asked with the failures of so many tau therapeutics. Is tau causal in disease? Conversely, is tau necessary for neuronal health and function? Most tau knockout mice are healthy. By reducing tau at a transcriptional level, the tau ASO trials are poised to answer this question, informing not only patient care but also our basic understanding of disease. The results of these trials, positive or negative, are likely to change our thinking and approach around tau therapeutics. Overall, with many compounds and approaches in development, the next decade will be an exciting time for the treatment of tauopathy.

## Author Contributions

C.L.D and A.L.B. conceptualized the article. C.L-D. wrote the initial draft and designed the figures. C.L-D. and A.L.B. reviewed and edited the manuscript.

## Declaration of competing interest

The authors declare the following financial interests/personal relationships which may be considered as potential competing interests: Adam Boxer reports a relationship with Alector LLC that includes: consulting or advisory and equity or stocks. Adam Boxer reports a relationship with Arvinas that includes: consulting or advisory and equity or stocks. Adam Boxer reports a relationship with Arkuda Therapeutics that includes: consulting or advisory and equity or stocks. Adam Boxer reports a relationship with Eli Lilly and Company that includes: consulting or advisory. Adam Boxer reports a relationship with Roche that includes: consulting or advisory. Adam Boxer reports a relationship with Merck & Co Inc that includes: consulting or advisory. Adam Boxer reports a relationship with Biogen that includes: funding grants. Adam Boxer reports a relationship with Eisai Inc that includes: funding grants. Adam Boxer reports a relationship with Regeneron Pharmaceuticals Inc that includes: funding grants. Adam Boxer reports a relationship with Janssen Pharmaceuticals Inc that includes: non-financial support. Adam Boxer reports a relationship with Denali Therapeutics Inc that includes: non-financial support. Adam Boxer reports a relationship with Oligomerix Inc that includes: consulting or advisory. Adam Boxer reports a relationship with Transposon Therapeutics Inc that includes: consulting or advisory. Adam reports a relationship with Amylyx Pharmaceuticals Inc that includes: consulting or advisory. Adam Boxer reports a relationship with Modalis that includes: consulting or advisory. Adam Boxer reports a relationship with GSK that includes: consulting or advisory. Adam Boxer has received research support from the National Institutes on Aging: NIH 1R01AG078457, U19AG063911, R01AG073482, R56AG075744, R01AG038791, RF1AG077557, R01AG071756, U24AG057437; Rainwater Charitable Foundation, Bluefield Project to Cure FTD, GHR Foundation, Alzheimer's Association, Association for Frontotemporal Degeneration, Gates Ventures, Alzheimer's Drug Discovery Foundation, UCSF Parkinson's Spectrum Disorders Center and the University of California Cures AD Program. If there are other authors, they declare that they have no known competing financial interests or personal relationships that could have appeared to influence the work reported in this paper.
